# Pneumorrachis and pneumocephalus associated with neck injury after stabbing

**DOI:** 10.1016/j.ijscr.2020.02.031

**Published:** 2020-02-19

**Authors:** Mehmet Huseyın Akgul

**Affiliations:** Kırıkkale Yüksek ihtisas State Hospital, Department of Neurosurgery, 71450, Kirikkale, Turkey

**Keywords:** Pneumorrrachis, Pnumocephalus, Neck injury, Stabbing

## Abstract

**Background:**

Trauma-related pneumocephalus and subcutaneous emphysema are relatively common, but pneumocephalus and pneumorrachis that occur without surgery are very rare. We present a case of pneumorrachis and pnemocephalus developing in the literature for the first time after stabbing from the anterior cervical region and providing improvement with conservative treatment.

**Case presentation:**

A 42-year-old male patient was brought to the emergency department after stabbed in the neck. Anteromedial injury of the sternocloid muscle was followed by two lacerations with active bleeding from the same site. The patient was unconscious (Glasgow coma score 8(E2, M4, V2). The patient was intubated. Bleeding foci and lacerations were repaired in the emergency. Cranial, cervical, thoracic and lumbar non-contrast computed tomography scans were performed. Moderate pneumocephalus was seen in the subarachnoid space in the anterior of the bilateral frontal lobe and in the suprasellar cistern region. Pneumorrachis was seen in C2-C7 levels of cervical spinal canal. The patient was pentotalized. 100% oxygen treatment for 6 h was given from the ventilator in intensive unit. After 72 h, cranial, cervical, thoracic and lumbar CT were performed. Pneumorrachis and pneumocephalus were fully recovered.

**Conclusion:**

Pneumorrachis is usually asymptomatic and is self-limiting. It is a radiological diagnosis and is not a clinical diagnosis. CT scan is considered the preferred diagnostic method for reliable and rapid detection of pneumorrachis. In case of coexistence, The physician should be alert to diagnose and treat the underlying cause for related injuries.In such cases, successful results can be obtained with hyper-oxy therapy (100% oxygen inhalation) and antibiotic prophylaxis without the need for surgical treatment.

## Highlights

• We present a case of pneumorrachis and pnemocephalus developing in the literature for the first time after stabbing from the cervical region.
• Pneumorrachis is usually asymptomatic and is self-limiting. It is a radiological diagnosis and is not a clinical diagnosis.
• CT scan is considered the preferred diagnostic method for reliable and rapid detection of pneumorrachis.
• In case of coexistence, The physician should be alert to diagnose and treat the underlying cause for related injuries.
• In such cases, successful results can be obtained with hyper-oxy therapy without the need for surgical treatment.

## 1 Background

Traumatic pneumocephalus and subcutaneous emphysema are relatively common. Pneumorrhachis refers to the presence of gas within the spinal canal (either intra- or extradural). It is rare. However, pneumocephalus and pneumorrachis seen without surgical intervention is a very rare condition [1]. Gordon and Hardman were the first to describe the intraspinal air phenomenon [2]. Pneumorrachis can also occur iatrogenically, secondary to pneumothorax, pneumomediastinum, pneumocephalus, subcutaneous emphysema, intestinal perforation, or postdiskectomy [3]. Very few cases of pneumorrachis associated with head trauma have been reported [4], but no case of pneumocephalus and pneumorrachis without fracture after neck trauma has been found in the literature.

In most cases, pneumorrachis is not associated with neurological symptoms [5]. Due to many etiologies, there is no definitive treatment. The first time in the literature, we present a case that provides an uneventful improvement with conservative treatment after pneumorrachis and pnemocephalus after stabbing from the anterior cervical region. This study was written in accordance with SCARE criteria [10].

## 2 Case presentation

**Fig. 1:**
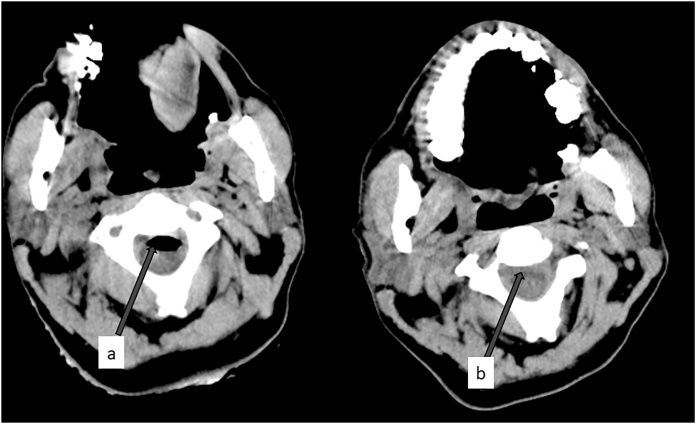
a. Noncontrast axial cervical CT shows pneumorachis in cervical C3 level (red arrow). b. Noncontrast axial cervical CT showed no improvement of pneumorrachis on the C3 level at 72 h (red arrow).

**Fig. 2:**
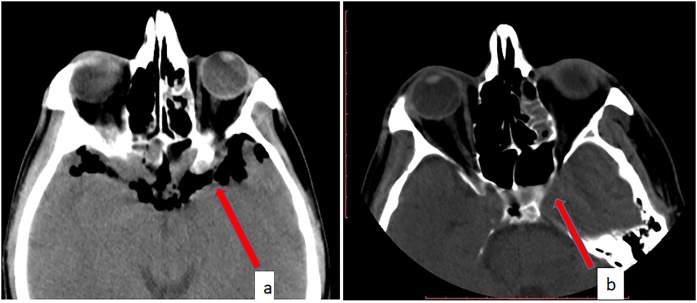
a. Noncontrast axial cranial CT showing pneumocephalus in the frontal region of the brain and in the subarachnoid space (red arrow). b. Noncontrast axial cranial CT showing disappearance of pneumocephalus at 72 h (red arrow).

A 42-year-old male patient was admitted to the emergency department after stabbed in the neck. After anteromedial injury of the sternocloid muscle, two lacerations with active bleeding from the same site (one of 5 cm in length and two on the paraspinal muscle of the left lumbar L2-4) were present. There were minimal contamination on the wound edges. The patient’s examination revealed stabbing in the neck and lumbar region. The scars on the lumbar region were related to the skin. However, neck injury was deeper. There was no other injury. On examination, the patient was unconscious (Glasgow coma score:8(E2 M4 V2)). Pulse rate was 65 per minute and blood pressure was 110/67 mmHg. Intravenous fluid and medical treatment were initiated. The lacerations in the emergency room were repaired under aseptic measures. Cranial, cervical, thoracic and lumbar non-contrast computed tomography (CT) scans were performed in the emergency department. Moderate pneumocephalus was seen in the subarachnoid spaces of the suprasellar cistern region in the anterior of the intracerebral bilateral frontal lobe. No cranial fracture was observed. The air levels were observed in the retrofaringeal region and in the neighborhood of the trachea until deep neck fascia in the cervical region. In addition, pneumorrachis was seen in cervical spinal canal C2-C7 levels. There was no evidence of fracture or subluxation in the cervical, thoracic and lumbar spine (Fig. 1a,b). The patient was intubated. Intravenous analgesic and antibiotic treatment was started. In addition, 100% oxygen from the ventilator was given for 6 h for the treatment of pneumocephaly and pneumorrachis. Four limbs were moving. There was no neurological deficit. It was pentotalized for 24 h due to loss of consciousness. Pentothal was stopped at 48th hour. In the sixth hour of pentotal discontinuity, the patient’s neurological examination revealed gks: 11 (E4 M6 V1e). He’s conscious. He left the ventilator and was then extubated. The patient’s neurological examination was normal after extubation (GKS: 15 (E4 M6 V5). After 72 h, cranial, cervical, thoracic and lumbar CT were performed. Pneumorrachis and pneumocephalus were completely resolved (Fig. 2a, b). He was discharged 4 days after admission. There were no problems in the late period. He was completely asymptomatic at three months follow-up.

## 3 Conclusion

Pneumorrachis is a rare radiological finding with different etiologies and possible pathways to the spinal canal. The case of intraspinal air is defined in various terms such as intraspinal pneumococcus, spinal epidural and subarachnoid pneumatosis or traumatic pneumomyelogram [[1], [2], [3]]. The term Pneumorrhachis was first described by Newbold et al. in 1987 [7]. There are only a few literature related to injuries. With the emergence of advanced imaging techniques such as computed tomography, such cases can be more easily identified. There are several reasons for the etiology of pneumorrachis. Pneumorrachis; Traumas (pneumothorax, pneumomediastinum, pneumocephalus, subcutaneous emphysema, intestinal perforation), high intrathoracic pressure and various respiratory diseases causing barotrauma, surgical or diagnostic procedures, malignancies, infections with gas-forming organisms and idiopathic diseases are known to occur due to various reasons [3]. In our case, we present the case of pneumocephalus and pneumorrachis after stabbing in the anterior region of the neck for the first time in the literature.

Goh et al. showed special attention to distinguish between air in the subarachnoid space and air in the epidural space, since both conditions had different clinical effects. Epidural air is generally harmless in itself, while subarachnoid pneumorrachis is an indication of serious injury and is commonly associated with pnemocephalus [5]. Traumatic subarachnoid pneumorrachis develops as secondary to pneumocephalus. However, in our case, it is seen that air passes through the cervical region to the cranium only after the neck injury without any cranial fracture with cranial dural rupture. The air can go into the cervical subarachnoid space or further when the intracranial compartment and the spinal canal are in contact with the trauma. This communication was demonstrated by Dandy, with the use of air as a negative contrast agent to the lumbar subarachnoid space for diagnostic pneumoencephalogram [8]. Pneumorrachis is usually asymptomatic and is primarily radiographic and is not a clinical diagnosis. A CT scan is considered the preferred diagnostic method for the reliable and rapid detection of pneumorrachis. However, it may be difficult to distinguish between epidural emphysema and subarachnoid pneumorrachis on CT. MRI or contrast-enhanced CT may be required for differentiation. Traumatic pneumorrachis is an indication of serious injury and its presence should alert the treating physician to carry out diagnostic work for related injuries. Due to its rare and diverse etiologies, there is no clear guideline for its treatment and is largely based on individual case reports. Cervical pneumorrachis with head trauma has not been reported to be associated with neurological deficits. Yousaf et al. reported radicular symptoms associated with pneumorrachis and reported that they successfully treated this case with oxygen [6]. The presence of traumatic subarachnoid pneumorachis indicates an obvious damage with a 25% risk of meningitis. Intravenous antibiotics can be initiated to prevent this possible complication. For important or persistent cerebrospinal fluid leakage, treatment with patch repair or temporary lumbar drainage can be performed [9]. If general anesthesia is required in such a patient, the anesthetist involved should not use inhaled nitrous oxide, causing an increase in intracranial pressure after spreading into air-filled cavities. In a few literature it is stated that additional oxygen therapy is used to facilitate air absorption [[3], [4], [5]].

This report aims to raise awareness about pneumorachis and pneumocephalus among neurosurgeons. Pneumorrachis is usually asymptomatic and self-limiting. In case of coexistence, the physician should warn the physician to diagnose related injuries and to treat the underlying cause. For these cases, successful results can be obtained with hyper-oxic therapy without surgery (with 100% oxygen inhalation) and prophylactic antibiotics.

Written informed consent was obtained from the patient for publication of this case report and accompanying images. A copy of the written consent is available for review by the Editor-in-Chief of this journal on request.

## Sources of funding

There is no funding sources of the study.

## Ethical approval

Ethics committee no approval was obtained. This studies is case report.

## Consent

İnformed consent is take to patient. Written informed consent was obtained from the patient for the publication of this case report and accompanying images. A copy of the written approval can be sent at any time by the Editor-in-Chief of this journal upon request.

## Author contribution

The article was written by a single author. For this reason, the author has done research, writing and sending the articles alone.

## Registration of research studies

NA.

## Guarantor

The writer was guarantor. The name is Mehmet Hüseyin Akgül.

## Provenance and peer review

Not commissioned, externally peer-reviewed.

## Declaration of Competing Interest

There is no financial disclosures of the authors.

Author Mehmet Hüseyin Akgül declares that he has no conflict of interest.
